# A Subanesthetic Dose of Isoflurane during Postconditioning Ameliorates Zymosan-Induced Neutrophil Inflammation Lung Injury and Mortality in Mice

**DOI:** 10.1155/2013/479628

**Published:** 2013-11-27

**Authors:** Hui Wang, Jing Fan, Nan-lin Li, Jun-tang Li, Shi-fang Yuan, Jun Yi, Ling Wang, Jiang-hao Chen, Yong-gang Lv, Qing Yao, Ting Wang, Yu-cai Wang, Rui Ling

**Affiliations:** ^1^Department of Vascular and Endocrine Surgery, Xijing Hospital, Fourth Military Medical University, Xi'an, Shaanxi 710032, China; ^2^Institute of Anal-Colorectal Surgery, No. 150 Central Hospital of PLA, Luoyang, Henan 451000, China; ^3^Department of Immunology, State Key Laboratory of Cancer Biology, Fourth Military Medical University, Xi'an, Shaanxi 710032, China; ^4^Department of Orthopaedic Surgery, Tangdu Hospital, Fourth Military Medical University, Xi'an, Shaanxi 710032, China

## Abstract

Anesthetic isoflurane (ISO) has immunomodulatory effects. In the present study, we investigated whether a subanesthetic dose of ISO (0.7%) protected against zymosan (ZY) induced inflammatory responses in the murine lung and isolated neutrophils. At 1 and 6 hrs after ZY administration intraperitoneally, ISO was inhaled for 1 hr, and 24 hrs later, lung inflammation and injury were assessed. We found that ISO improved the survival rate of mice and mitigated lung injury as characterized by the histopathology, wet-to-dry weight ratio, protein leakage, and lung function index. ISO significantly attenuated ZY-induced lung neutrophil recruitment and inflammation. This was suggested by the downregulation of (a) endothelial adhesion molecule expression and myeloperoxidase (MPO) activity in lung tissue and polymorphonuclear neutrophils (b) chemokines, and (c) proinflammatory cytokines in BALF. Furthermore, ZY-induced nuclear translocation and DNA-binding activity of NF-**κ**B p65 were also reduced by ISO. ISO treatment inhibited iNOS expression and activity, as well as subsequent nitric oxide generation. Consistent with these *in vivo* observations, *in vitro* studies confirmed that ISO blocked NF-**κ**B and iNOS activation in primary mouse neutrophils challenged by ZY. These results provide evidence that 0.7% ISO ameliorates inflammatory responses in ZY-treated mouse lung and primary neutrophils.

## 1. Introduction

Multiple organ dysfunction syndrome (MODS) leads to high morbidity and mortality rates in the intensive care unit and is one of the most urgent and challenging public health problems worldwide [1, 2]. The lung is frequently the first organ that fails during the development of this syndrome. However, the mechanism of lung injury induced by inflammation remains to be determined, and the therapeutic regimen requires further investigation.

Zymosan-induced generalized inflammation (ZIGI) mouse model can reproduce many characteristics of human MODS, which is adopted by many research groups [3, 4]. Several reports have shown that the onset of ZY-induced inflammatory response in mouse lung is associated with the gas exchange barrier and that it culminates with maximal neutrophil accumulation, exudate formation, and proinflammatory cytokines production [5–7]. ZY is recognized by toll-like receptor 2 (TLR-2) on immune cells (e.g., neutrophils), which subsequently trigger signal cascade for nuclear factor-*κ*B (NF-*κ*B) activation [8]. NF-*κ*B activation is required for maximal expression of many proinflammatory cytokines and chemokines and iNOS involved in the pathogenesis of acute lung injury [9].

ISO is a widely used inhaled anesthetic, which exerts protective properties mainly through antioxidant and anti-inflammatory properties [10, 11]. Several studies have demonstrated that the anti-inflammatory activity of ISO at anesthetic concentration (1.2%–2.5%) is associated with (A) ameliorated lung dysfunction and mortality [12], (B) decreased proinflammatory cytokine and chemokine release, (C) decreased polymorphonuclear neutrophil infiltration [13], and (D) diminished NF-*κ*B and inducible nitric oxide synthase-NO (iNOS-NO) pathway activation [12, 14]. However, ISO at clinical anesthetic dose has adverse effects for critically ill patients, who cannot tolerate its hemodynamic effects that include vasodilation, myocardial depression, and bradycardia [15]. ISO at less than 1% for sedation weakly interferes with hemodynamics, which is more beneficial for critically ill patients in the intensive care unit [16, 17]. Our recent study demonstrated that ISO at a subanesthetic dose (0.7%) results in suppression of inflammatory responses via antioxidant activity in ZY-induced lung injury [18]. However, it is not known whether the inhibition of ZY-induced pulmonary injury in mice by subanesthetic doses of ISO is promoted by its anti-inflammatory properties. The purpose of this study was to investigate how the suppression of the inflammatory response by 0.7% ISO contributes to its ability to attenuate ZY-induced inflammatory lung injury in mice.

## 2. Materials and Methods

### 2.1. Reagents

All reagents were purchased from Sigma-Aldrich (St. Louis, MO, USA) unless otherwise stated. NF-*κ*B activation inhibitor (NAI) and ISO were obtained from Calbiochem (Darmstadt, Germany) and Baxter (Baxter Healthcare Corporation, Deerfield, IL), respectively. All suspensions were freshly made before use.

### 2.2. Animals and Treatments

Male BALB/C mice (8 weeks old and weighing 22–25 g) were used in this study. Animal procedures were approved by the Ethics Committee for Animal Experimentation of Fourth Military Medical University. Euthanasia by pentobarbital was consistent with the AVMA Guidelines on Euthanasia, June 2007.

An inflammation-associated lung injury model was established by aseptic intraperitoneally (IP) injection of ZY (25 mg/mL suspended in normal saline (NS)) into mice, at a dose of 1 g/kg of body weight, as previously described [18, 19]. The animals were placed in a sealed plexiglass chamber with inflow and outflow outlets. The same volume of NS was injected through the same route as the sham control. Mice were exposed to ISO via inhalation as the previous study [18, 19]. Briefly, ISO was delivered by air into the chamber through a tube at a rate of 4 L/min. The flow rate of ISO was accurately and real-time controlled by regulation of Anesthetic Vaporizers (Harvard apparatus, USA). The concentration of ISO in the outflow hose of the chamber was continuously monitored with a gas analyzer (Brüel & Kjae, Naerum, Denmark) and maintained at 0.7% during the treatment. The concentration of oxygen in the chamber was maintained at 21% by using supplemental oxygen and continuously monitored with a gas analyzer (Medical Gas Analyzer LB-2, Model 40 M; Beckman, Fullerton, CA). Carbon dioxide was removed from the chamber gases with baralyme (Allied Healthcare Products, Inc., St. Louis, MO). The animals without ISO treatment were exposed to room air (RA) in the chamber as the vehicle control.

### 2.3. Neutrophil Isolation and Culture

Neutrophils were isolated from peripheral venous blood of healthy mice using an anti-Ly-6G MicroBead Kit (Miltenyi Biotec, Germany) according to the manufacturer's protocol. Isolated neutrophils (5 × 10^6^/mL) were cultured as previously described [20]. Prior to all experiments, >99% of cells were determined viable by Live/Dead violet (Invitrogen, Carlsbad, CA).

### 2.4. Experimental Design

**Figure 1:**
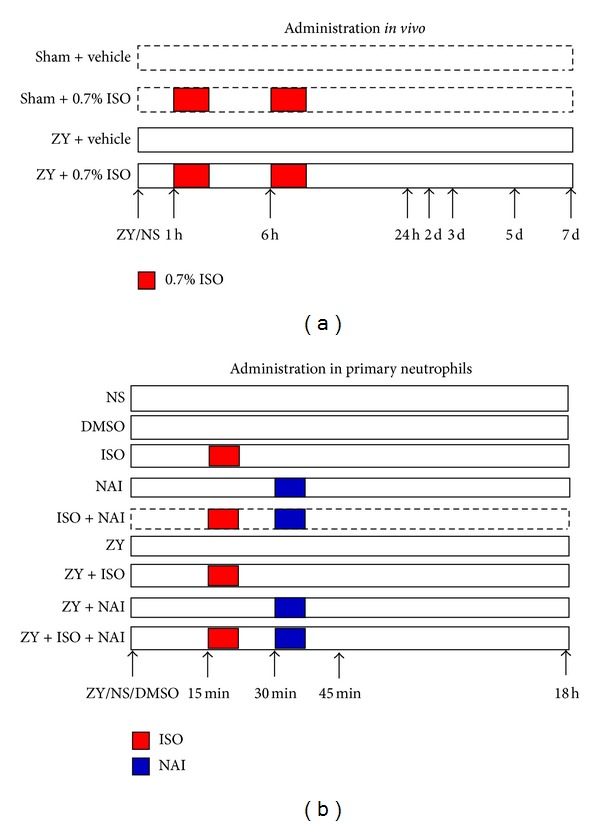
Schematic diagram depicting the experimental designs for this study. (a) Four treatment groups were used for *in vivo* experiments. At 1 and 6 hrs after NS (sham) or ZY injection, mice were subjected to inhalation of 0.7% ISO or RA (vehicle) for a 1 hr duration. BALF and lung tissues were harvested for a series of assays at 24 hrs following NS or ZY administration. (b) Test and control groups for 0.7% ISO treatment of isolated neutrophils. At 45 mins or 18 hrs after single or combined treatments with ZY, ISO or NF-*κ*B activation inhibitor (NAI) in neutrophils, assays for NF-*κ*B DNA-binding activity, iNOS activity, and NO formation were performed. DMSO was assayed as a solvent control. For more detailed descriptions, see Section 2.

For *in vivo* studies, eighty mice were randomly allocated as follows (*n* = 20  per group; Figure 1(a)). (1) ZY + vehicle group: mice were given an IP injection of ZY (1 g/kg, dissolved in NS solution), followed by inhalation of RA (vehicle) for 1 h starting at 1 h and 6 h after ZY administration. (2) ZY + 0.7% ISO group: no differences from the ZY + vehicle group, except for 1 h inhalation of ISO starting at 1 h and 6 h instead of RA after ZY administration. (3) Sham + vehicle group: no differences from the ZY + vehicle group, except for administration with NS (sham) instead of ZY. (4) Sham + 0.7% ISO group: identical to the sham + vehicle group, except for 1 h inhalation of ISO starting at 1 h and 6 h after NS (Sham) administration. At 24 hrs after administration of ZY, animals were assessed for ZY-induced lung injury. In another set of experiments, animals (*n* = 20  each group) were randomly assigned and monitored for survival for 7 days after ZY/ISO or NS/ISO administration.

For *in vitro* studies, primary mouse neutrophils were plated in 6-well plates and treated with the following reagents (Figure 1(b)): sterile NS, DMSO, ISO (0.15 mM, equal to 0.7%), NF-*κ*B activation inhibitor (NAI, 10 *μ*M; dissolved in DMSO), ZY (1.5 mg/mL), ISO for 15 mins following ZY (ZY + ISO), NAI for 15 mins following ISO (ISO + NAI), NAI for 15 mins following ZY ( ZY + NAI ), or NAI for 15 mins following ISO for 15 mins and then following ZY (ZY + ISO + NAI). Assays for NF-*κ*B or iNOS expression and activity and iNOS-derived NO formation were performed 45 mins or 18 hrs after treatment with ZY/NS/DMSO, respectively.

### 2.5. Histologic Examination

Lungs were harvested for observing morphologic alterations at 24 hrs after ZY or NS administration. The subjects were fixed with 10% formalin for 8 hrs at room temperature, embedded in paraffin, and sectioned at 4 *μ*m thickness. After deparaffinization and rehydration, the sections were sequentially stained with hematoxylin and eosin. Histologic changes were evaluated by two independent pathologists, who had no knowledge of the treatment regimen received by each respective animal. The degree of lung injury was scored on a subjective scale ranging from 0 to 3; 0 = absence, 1 = mild, 2 = moderate, and 3 = severe. The ranging scale was used for each of histologic features: edema, hyperemia and congestion, neutrophil margination and tissue infiltration, intra-alveolar hemorrhage and debris, and cellular hyperplasia. The final score will be the adding of the single evaluation [21].

### 2.6. Wet/Dry Weight Ratio

To quantify the magnitude of pulmonary edema, we evaluated lung wet/dry (W/D) weight ratio at 24 hrs after NS or ZY administration. The harvested wet lung was weighed and then placed in an oven for 24 hrs at 80°C and weighed when it was dried. The ratio of wet lung to dry lung was calculated [22].

### 2.7. Protein Leakage

Total protein concentration in the BALF was determined using a standard commercial kit (Bio-Rad Laboratories, Hercules, CA).

### 2.8. pH/Blood Gases Analysis

At 24 hrs after NS or ZY administration, blood samples were taken and centrifuged (1500 g for 5 min at room temperature) to separate plasma. For the evaluation of acid-base balance and blood gas analysis (indicators of lung function), arterial blood levels of pH, PaO_2_, and PCO_2_ and  HCO_3_^−^  were determined by pH/blood gases analyzer as previously described [23].

### 2.9. BALF Collection and Cell Counts

At 24 hrs after administration of ZY or NS, BALF collection was performed by the methods described previously [24]. The mice were anesthetized with pentobarbital, tracheas were cannulated after exsanguination, and lungs were gently washed with 2 mL of PBS. The amount of exudate was calculated by subtracting the volume injected (2 mL) from the total volume recovered. BALF samples were centrifuged at 500 g at 4°C for 12 mins, and the supernatant was stored at − 70°C for subsequent analysis of protein and cytokine levels. Furthermore, cell pellets were resuspended in 1 mL of PBS, and the number of total cells was determined using a hemocytometer (Beckman Coulter, Inc). For differential cells counts, cytospin slides were prepared and stained with Diff-Quick [25], and every kind of cell was identified by a certified laboratory technologist in a blinded fashion.

### 2.10. Measurement of Lung MPO Activity

Myeloperoxidase (MPO) activity was measured as an indicator of neutrophil infiltration into the lung tissue as previously described [26]. At 24 hrs after ZY or NS injection, all animals (*n* = 10  for each group) were sacrificed with pentobarbital. Lungs were obtained and perfused with cold PBS to remove all blood, and homogenated lung supernatants were prepared to detect the activity of MPO. MPO activity was defined by the change in absorbance measured by spectrophotometer (DU 640B; Beckman) at 590 nm and expressed in unit per gram weight of wet tissue. The activity of MPO was measured by using commercial kits purchased from Cayman Chemical Company.

### 2.11. Measurement of Cytokine and Chemokine Production

At 24 hrs after ZY or NS injection, the cytokines and chemokines levels in BALF were measured using commercially available enzyme-linked immunosorbent assay (ELISA) kits (mouse TNF-*α*, IL-1*β*, IL-6, high-mobility group box-1 (HMGB-1), keratinocyte-derived chemokine (KC), macrophage inflammatory protein-1*α* (MIP-1*α*), macrophage inflammatory protein-2 (MIP-2), and monocyte chemoattractant protein-1 (MCP-1) ELISA kits are from R&D Systems, Minneapolis, MN). The optical density (OD) was measured on an ELISA plate scanner (CA94089, Molecular Devices, Sunnyvale, Canada). All experiments were performed according to the manufacturers' instructions [27].

### 2.12. Determination of iNOS Enzyme Activity

Measuring iNOS activity by monitoring the conversion of arginine to citrulline was a standard assay as described previously [28]. At the predetermined time points (see experimental design or Figure 1), a aliquot of homogenated lung tissue or neutrophil lysate was incubated with L-[^3^H] arginine accompanied with the essential substrates and cofactors (tetrahydrobiopterin, nicotinamide adenine dinucleotide phosphate, flavin adenine dinucleotide, etc.), and the production of L-[^3^H] citrulline was calculated by liquid scintillation counting. For the quantification of iNOS activity, ethylenediamine tetraacetic acid (EDTA) and ethylene glycol tetraacetic acid (EGTA) were sequentially added to the incubation buffer. An appropriate blank was needed as a reaction including 1 mM L-NAME (competitive iNOS inhibitor) to exclude the effect from the background of the nonspecific metabolism of L-arginine and the similar description sees our previous study [19]. iNOS activity in the citrulline assay was determined by the L-NAME inhibitable degree in the EDTA-EGTA sample, and its expression was measured using Units (1 Unit = 1 pmol L-citrulline/mg protein/min).

### 2.13. Measurement of Nitrite Concentration

Production of nitrite (NO_2_^−^), an indicator of NO synthesis, was assessed using a colorimetric reaction with the Griess reagent [29]. At the predetermined time points (see experimental design or Figure 1), BALF or the neutrophil culture media was collected and mixed with an equal (1 : 1) volume of Griess reagent (0.1% N-(1-naphthyl) ethylenediamine dihydrochloride, 1% sulfanilamide, and 2.5% H_3_PO_4_). A 96-well microplate reader (Spectra MAX 340PC, Molecular Devices) was used to measure the absorbance at 540 nm; data were analyzed using Softmax Pro software. Sodium nitrite was dissolved in double-distilled water then used as standards.

### 2.14. Western Blot Analysis

At 24 hrs after ZY or NS injection, cytosolic and nuclear extracts of homogenated lung tissue were prepared with a nuclear extract kit (Active Motif, Carlsbad, CA). According to the manufacturer's instructions, all standards and samples were run in triplicate [29]. NF-*κ*B p65 levels were quantified in nuclear fractions. All other protein levels were quantified in cytosolic fractions. The ultimate two extracts (cytosolic and nuclear protein) were boiled, separated by sodium dodecyl sulfate polyacrylamide gel electrophoresis (SDS-PAGE), electrotransferred onto nitrocellulose membranes, and then immunoblotted with rabbit anti-iNOS polyclonal antibody (pAb) (Millipore, Temecula, CA, USA), rabbit anti-NF-*κ*B pAb, and rabbit anti-I*κ*B pAb (Santa Cruz Biotechnology, CA, USA). Equivalent sample loading was confirmed by probing with mouse anti-*β*-actin monoclonal antibody (mAb) and rabbit anti-laminin B mAb (Sigma, CA, USA). Detection was performed with the enhanced chemiluminescence assay kit (Pierce, Rockford, IL, USA).

### 2.15. NF-*κ*B DNA-Binding Activity Assay

At the predetermined time points (see experimental design or Figure 1), the DNA-binding activity of NF-*κ*B in lung tissues and neutrophils was quantified using the TransAM NF-*κ*B p65 transcription factor assay kit (Active Motif, Carlsbad, CA). The nuclear extracts of lung tissues were prepared with a nuclear extract kit (Active Motif). According to the manufacturer's instructions, all standards and samples were run in duplicate [30].

### 2.16. Quantitative Real-Time Reverse Transcriptase (RT)-PCR

At the predetermined time points (see experimental design or Figure 1), total RNA from lung tissue or neutrophils was isolated and extracted with TRIzol Regant (Invitrogen, Carlsbad, CA, USA) following the instructions of the manufacturer. For quantitative real-time reverse transcriptase (RT)-PCR analysis, cDNA was synthesized from total RNA with SuperScript Reverse Transcriptase kit (Invitrogen). Quantitative real-time RT-PCR reaction was performed with iQ5 Real-Time PCR Detection System (Bio-Rad, Hercules, CA, USA). The primers had the following sequences: for iNOS, forward 5′-AACGGAGAA CGTTGGATTTG-3′ and reverse 5′-CAGCACAAGGGGTTTT CTTC-3′; for E-selectin, forward 5′-TCTGGACCTTTCCAAAATGG-3′ and reverse 5′-TGCAAGCTAAAGCCCTCATT-3′; for VCAM-1, forward 5′-TGGAGGAAATGGGCATAAAG-3′ and reverse 5′-CAGGATTTTGGGAGCTGGTA-3′; for ICAM-1, forward 5′-CGAAGGTTCTTCTGAGC-3′ and reverse 5′-GTCTG CTGAGACCCCTCTTG-3′; for *β*-actin, forward 5′-TGAGAGGGAAATCGTGCGTG-3′ and reverse 5′-TTGCTGATCCACA TCTGCTGG-3′. The PCR settings were as follows: initial denaturation at 95°C was followed by 25 cycles of amplification for 10 s at 95°C and 20 s at 56°C, with subsequent melting curve analysis, increasing the temperature from 72 to 95°C. Quantification of gene expression was calculated relative to *β*-actin.

### 2.17. Immunohistochemistry

Immunohistochemistry was performed as previously described [31]. At 24 hrs after ZY or NS injection, the lung tissues were fixed in 10% PBS-buffered formalin, and 5 *μ*m sections were prepared from paraffin-embedded tissues. After deparaffinization, endogenous peroxidase was blocked with 0.3% (volume/volume [v/v]) hydrogen peroxide in 60% (v/v) methanol for 30 mins. The sections were permeabilized with 0.1% (v/v) PBS-buffered Triton X-100 for 20 mins. Incubate the section in 3% (v/v) normal goat serum in PBS for 20 mins to minimize the nonspecific adsorption. Endogenous biotin or avidin binding sites were blocked by sequential incubation for 15 mins with avidin and biotin (BD biosciences, CA, USA). The sections were then incubated overnight with rabbit anti-iNOS mAb (BD biosciences, CA, USA, 1 : 500 in PBS, v/v) or with control solutions. Controls included buffer alone or nonspecific purified rabbit immunoglobulin G. A biotin-conjugated specific secondary anti-immunoglobulin G and avidin-biotin peroxidase complex were used to detect the specific labeling. To verify the binding specificity for iNOS, some sections were also incubated with primary antibody only (no secondary antibody) or with secondary antibody only (no primary antibody). In these situations, no positive staining was found in the sections indicating that the immunoreactions were positive in all the experiments carried out.

### 2.18. Statistical Analyses

With the exception of histologic scores, all values are expressed as mean ± SD. Survival data were calculated by the Fisher exact probability test and expressed as percentages. The histologic scores were analyzed with the Kruskal-Wallis test followed by the Mann-Whitney *U* test with a Bonferroni correction. The intergroup differences were tested by one-way analysis of variance followed by a least-significant-difference (LSD) *t* test for multiple comparisons. GraphPad statistical software (GraphPad Software, Inc., San Diego, CA) was used to perform the data analysis. Values of  *P* < 0.05  were considered statistically significant.

## 3. Results

### 3.1. Subanesthetic ISO Treatment Improves the Survival Rate in ZY-Challenged Mice

**Figure 2:**
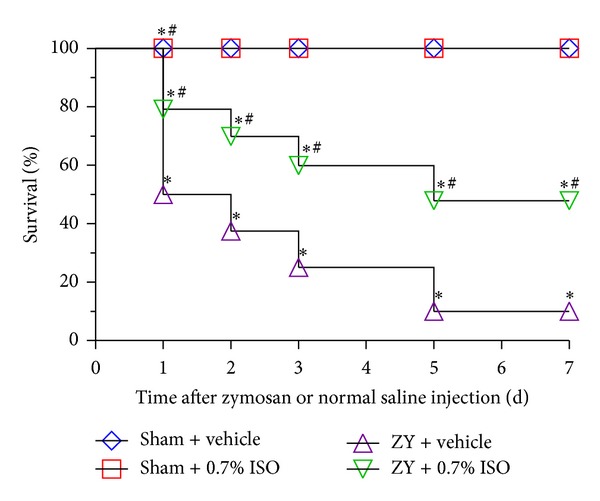
Effect of 0.7% ISO treatment on mortality of ZY-challenged mice. Mice were treated with RA (vehicle) or ISO inhalation for 1 hr at 1 and 6 hrs after NS (sham) or ZY injection. The survival percentage was calculated each day for 7 days following initial treatment (*n* = 20  per group).  *P < 0.05  versus sham + vehicle;  ^#^ *P* < 0.05  versus ZY + vehicle.

As expected, all the mice from the Sham + vehicle and Sham + ISO groups survived. And no significant differences existed in the two groups. However, the 7-day survival rate for ZY-challenged mice was 10% (Figure 2). Inhalation of 0.7% ISO for 1 hr starting at 1 and 6 hrs after ZY injection improved the 7-day survival rate from 10% to 50% (Figure 2). This suggests that 0.7% ISO treatment can significantly reduce the mortality of ZY-challenged mice.

### 3.2. Subanesthetic ISO Treatment Alleviates Lung Injury in ZY-Challenged Mice

**Figure 3:**
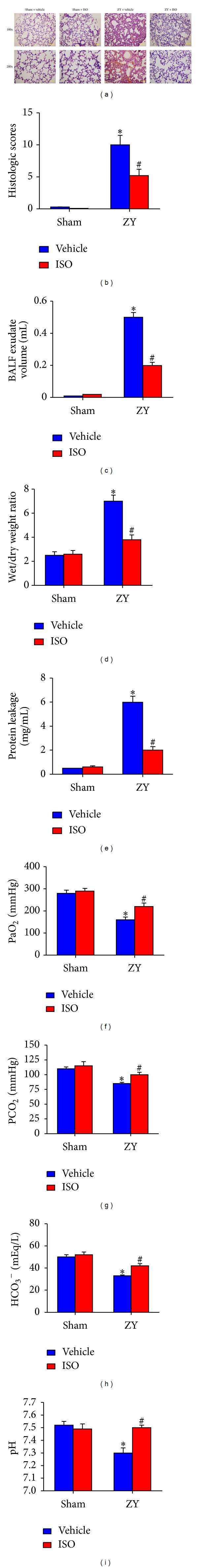
ISO at 0.7% preserved lung architecture and normalized lung function in ZY-challenged mice. (a) Lung morphology as assessed by hematoxylin and eosin staining (top, 100x magnification; bottom, 200x magnification). (b) Histologic scoring for the treatment groups in Figure (a). ((c)–(e)) Measurement of markers of lung injury: BALF exudate volume (c), lung wet/dry weight ratio (d), and protein leakage (e). ((f)–(i)) Measurement of markers of lung intactness: PaO_2_ (f), PCO_2_ (g),  HCO_3_^−^  (h), and pH (i). The animals were treated as shown in Figure 1(a). Data represent means ± SD (*n* = 10  mice per group).  *P < 0.05  versus sham + vehicle ^#^ *P* < 0.05  versus ZY + vehicle.

Histology studies showed that ZY-challenged mice have significant lung injury characterized by alveolar wall thickening, infiltration of neutrophils into lung interstitium, consolidation, and alveolar hemorrhage (*P* < 0.05; Figures 3(a) and 3(b)) but that ISO treatment resulted in a reduction of inflammatory response and a marked improvement in lung architecture (*P* < 0.05; Figures 3(a) and 3(b)). No histologic alteration was observed in the lung from sham-treated mice (*P* < 0.05; Figures 3(a) and 3(b)). ZY-challenged mice also showed marked increases in exudate volume, wet-to-dry weight ratio, and protein leakage as compared with the sham group, and these increases in indicators of lung injury were significantly reduced by ISO treatment (*P* < 0.05; Figures 3(c)–3(e)). Moreover, ZY administration in mice led to significant reduction in the levels of markers for lung function, including the arterial levels of PaO_2_, PCO_2_,  HCO_3_^−^, and pH as compared to sham mice, and these values were normalized by ISO treatment (Figures 3(f)–3(i)). These results suggest that 0.7% ISO treatment mitigates lung injury caused by ZY.

### 3.3. Subanesthetic ISO Treatment Reduces ZY-Induced Activation of NF-*κ*B

**Figure 4:**
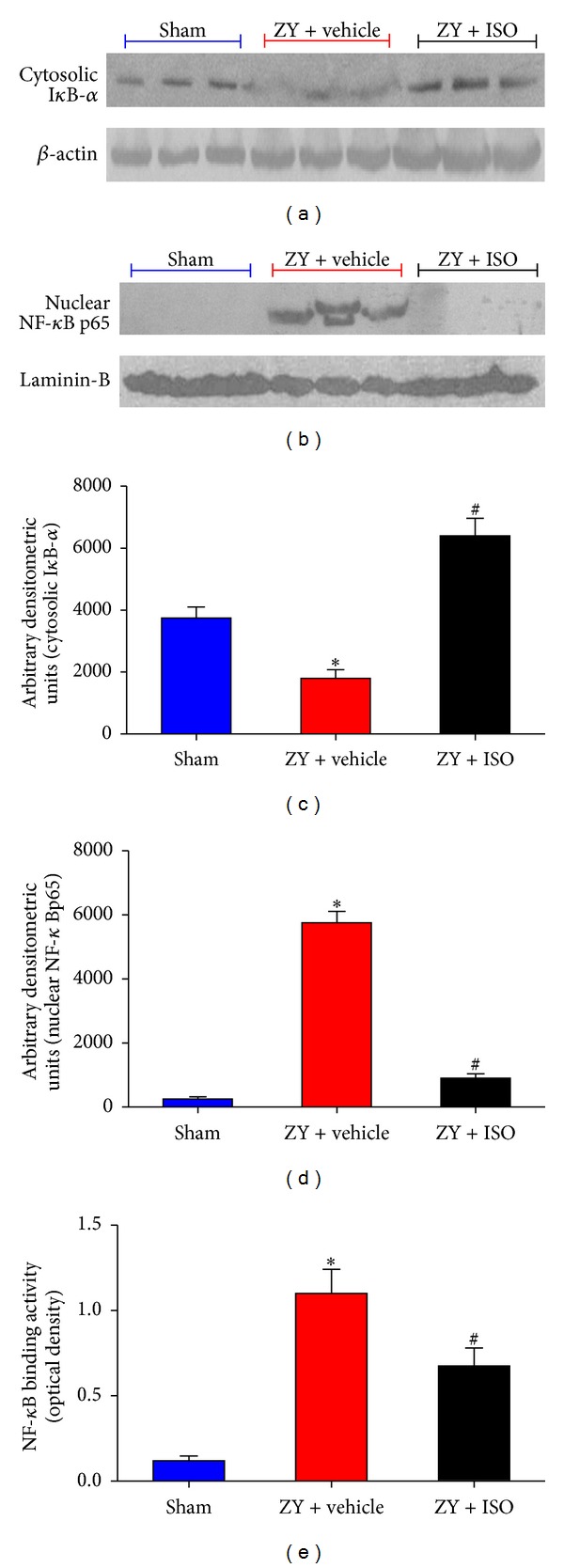
NF-*κ*B activation caused by ZY in lung at 24 hrs was reduced by 0.7% ISO treatment. Representative western blot for cytoplasmic I*κ*B degradation (a) and nuclear translocation of NF-*κ*B p65 (b). Results were quantified as arbitrary densitometry units ((c) and (d)). (e) NF-*κ*B DNA-binding activity as assayed by optical density. The animals were treated as shown in Figure 1(a). Data represent means ± SD (*n* = 10  mice per group).  *P < 0.05  versus sham + vehicle;  ^#^ *P* < 0.05  versus ZY + vehicle.

NF-*κ*B is a critical transcription factor required for maximal expression of pro-inflammation cytokines in ZY-induced shock [9]. To investigate the cellular mechanisms whereby 0.7% ISO treatment attenuated ZY-induced lung injury, we evaluated I*κ*B-*α* degradation and the nuclear accumulation of NF-*κ*B p65 as markers of NF-*κ*B activation. I*κ*B-*α* levels in lung tissues were substantially reduced in ZY-treated mice compared to the sham group, while treatment with ISO prevented ZY-induced I*κ*B-*α* degradation (*P* < 0.05; Figures 4(a) and 4(c)). Furthermore, the nuclear translocation of NF-*κ*B p65 was dramatically elevated 24 hrs after ZY administration as compared with the sham group, but this elevation was reversed by ISO treatment (*P* < 0.05; Figures 4(b) and 4(d)). Assays for NF-*κ*B DNA binding activity in pulmonary tissue further supported these findings (*P* < 0.05; Figure 4(e)). These data demonstrate that ZY results in lung NF-*κ*B activation in ZY-challenged mice and that this activation is blocked by ISO treatment.

### 3.4. Effect of Subanesthetic ISO Treatment on Cytokine Expression

**Figure 5:**
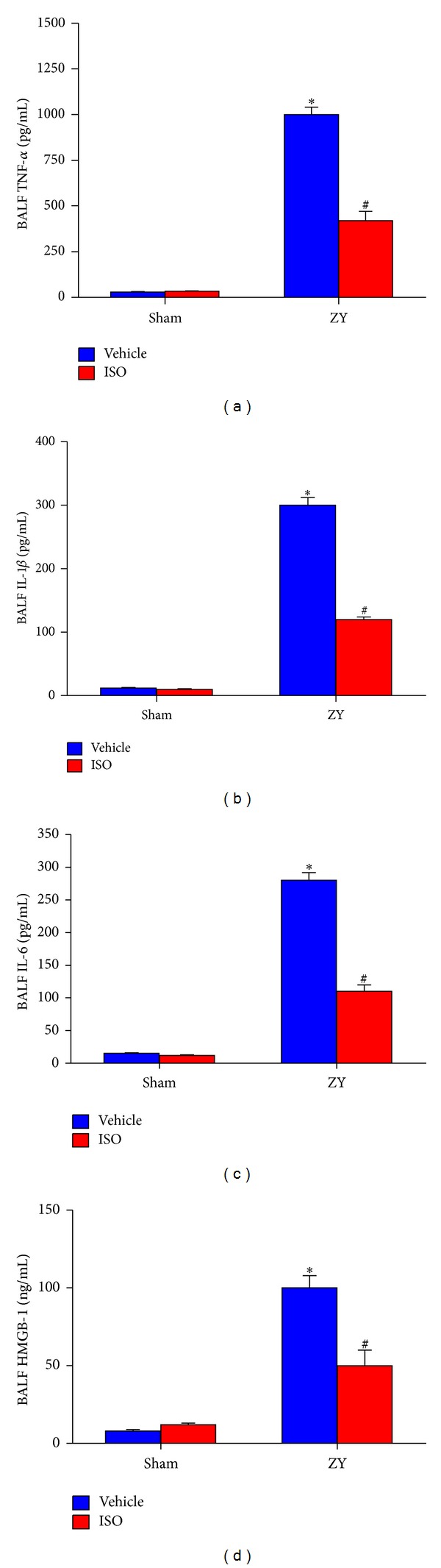
Effect of 0.7% ISO treatment on cytokine production in BALF in ZY-treated mice at 24 hrs. The following inflammatory indicators in BALF were measured by ELISA: (a) TNF-*α*; (b) IL-1*β*; (c) IL-6; and (d) HMGB-1. The animals were treated as shown in Figure 1(a). Data represent means ± SD (*n* = 10  mice per group).  *P < 0.05  versus sham + vehicle;  ^#^ *P* < 0.05  versus ZY + vehicle.

The levels of the proinflammatory cytokines tumor necrosis factor *α* (TNF-*α*), interleukin 1*β* (IL-1*β*), interleukin 6 (IL-6), and high-mobility group box-1 (HMGB-1) in the BALF were significantly increased 24 hrs after ZY administration in mice compared with those of the sham groups (*P* < 0.05; Figure 5). However, ISO treatment dramatically attenuated proinflammatory cytokine production (*P* < 0.05; Figure 5). These results indicate that ISO treatment reduces proinflammatory cytokine levels in the BALF from ZY-challenged mice.

### 3.5. Subanesthetic ISO Treatment Suppresses ZY-Induced iNOS/NO Production

**Figure 6:**
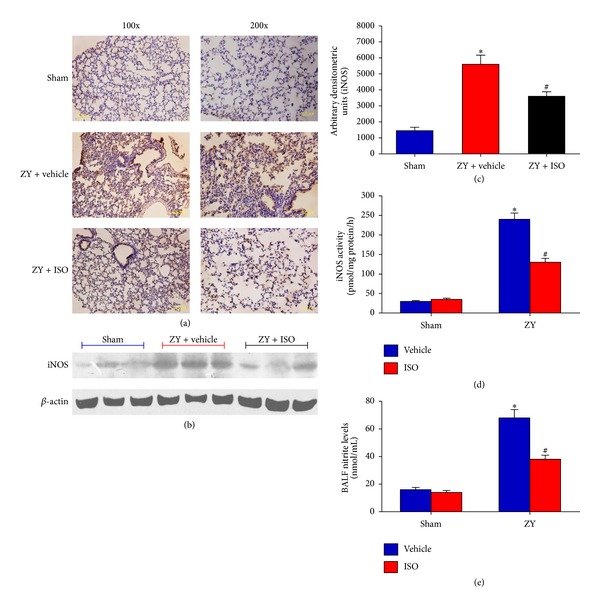
Effects of 0.7% ISO treatment on iNOS/NO production in ZY-treated lung at 24 hrs. iNOS protein expression was assayed by immunohistochemistry (a) and western blot (b). (c) The average iNOS staining from western blots was quantified by arbitrary densitometry units. (d) iNOS enzyme activity in the four treatment groups. (e) Nitrite levels in the four treatment groups as an indicator of NO formation. The animals were treated as shown in Figure 1(a). Data represent means ± SD (*n* = 10  mice per group).  *P < 0.05  versus sham + vehicle;  ^#^ *P* < 0.05  versus ZY + vehicle.

NO mediates inflammatory response, which produces high levels of this endogenous free radical upon induction of iNOS by inflammatory stimulus [32]. Additionally, iNOS activity is regulated by NF-*κ*B in mice after ZY insult [33]. A substantial increase in iNOS expression was detected by immunohistochemistry (*P* < 0.05; Figure 6(a)) and western blot (*P* < 0.05; Figures 6(b) and 6(c)) in lung tissue 24 hrs after ZY administration compared with the sham group. ISO treatment significantly reduced pulmonary iNOS expression (*P* < 0.05; Figures 6(a)–6(c)). In addition, both iNOS activity and BALF  NO_2_^−^  levels were substantially increased in ZY-treated mice compared with the sham-operated mice, and this increase was significantly reduced by ISO treatment (*P* < 0.05; Figures 6(d) and 6(e)). The data suggests that ISO treatment attenuates pulmonary iNOS/NO production in ZY-challenged mice.

### 3.6. Subanesthetic ISO Treatment Inhibits ZY-Induced Neutrophil Recruitment into the Lung

ZY-treated mice exhibited a marked increase in total cells and polymorphonuclear neutrophils in the BALF at 24 hrs, and this increase was significantly reduced by ISO treatment (Table 1). However, ZY had no significant effect on recruitment of lymphocytes, macrophages, and eosinophils into the lung.

**Figure 7:**
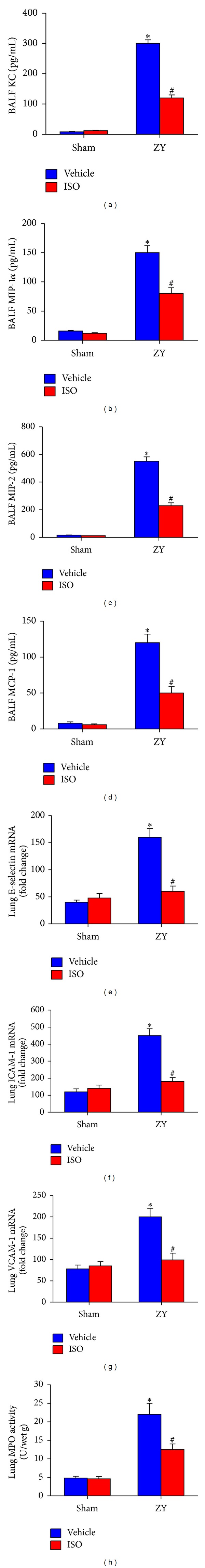
Effects of 0.7% ISO treatment on chemokine production, endothelial adhesion protein expression, and neutrophil recruitment in ZY-treated lung at 24 hrs. Levels of the following chemokines in BALF were measured by ELISA: (a) keratinocyte-derived chemokine (KC); (b) macrophage inhibitory protein-1*α* (MIP-1*α*); (c) macrophage inhibitory protein-2 (MIP-2); (d) monocyte chemoattractant protein-1 (MCP-1). The mRNA expression of the following endothelial adhesion molecules in lung tissue from all groups was measured by qPCR: (e) E-selectin; (f) ICAM-1; (g) VCAM-1. Lung MPO activity is shown in (h). The animals were treated as shown in Figure 1(a). Data represent mean ± SD (*n* = 10  mice per group).  *P < 0.05  versus sham + vehicle;  ^#^ *P* < 0.05  versus ZY + vehicle.

Chemokines play a critical role in neutrophil infiltration into the lung upon inflammation stimulus [34]. Our studies showed that the levels of KC, MIP-1*α*, MIP-2, and MCP-1 in BALF were dramatically elevated 24 hrs following ZY injection compared with sham groups, but this elevation was significantly reduced by ISO treatment (*P* < 0.05; Figures 7(a)–7(d)). Because neutrophil influx is dependent on the activation of endothelial cells [35], we examined the effect of ISO on the expression of endothelial adhesion molecules in lung tissue from ZY-treated mice. Compared to sham-treated animals, ZY increased the pulmonary mRNA levels of E-selectin, intercellular adhesion molecule (ICAM)-1, and vascular cell adhesion molecule (VCAM)-1, and this increase was also significantly reduced by ISO treatment (*P* < 0.05; Figures 7(e)–7(g)). We also examined lung MPO activity as an indicator of neutrophil infiltration 24 hrs after ZY/ISO administration compared with sham treatment. The lung MPO activity of ZY-challenged mice dramatically increased, and this increase was significantly attenuated by ISO treatment (*P* < 0.05; Figure 7(h)). Taken together, these results indicate that ISO treatment inhibits ZY-induced neutrophil recruitment into the lung by regulating the expression of several extravasation-associated proteins.

### 3.7. Subanesthetic ISO Treatment Inhibits ZY-Induced NF-*κ*B Activation and iNOS Activity and NO Formation *In Vitro*

**Figure 8:**
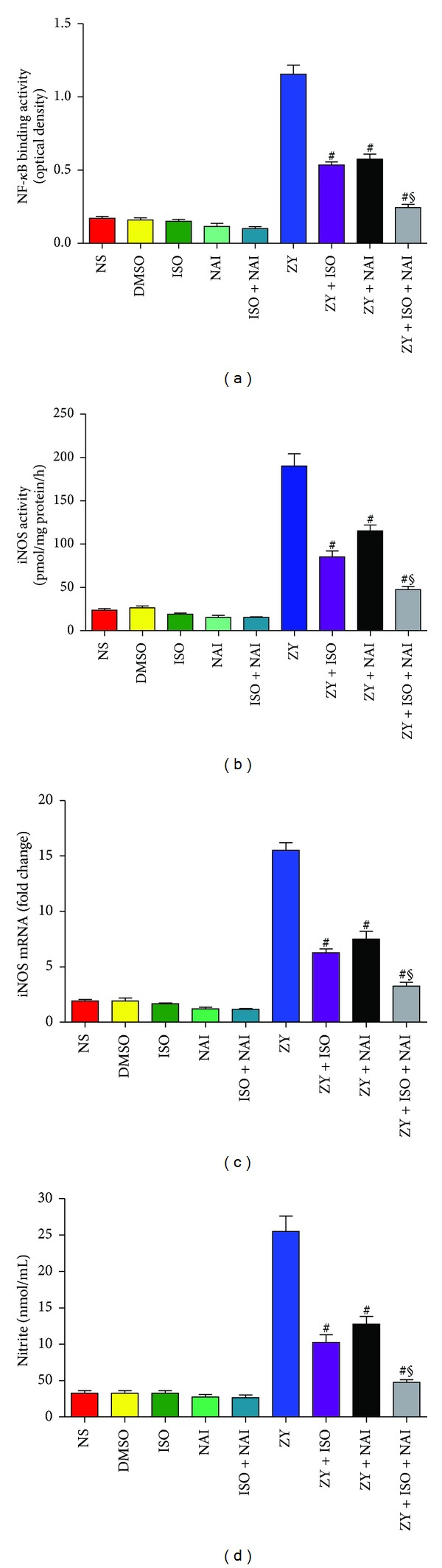
ISO at 0.7% inhibited ZY-induced NF-*κ*B activation, iNOS activity, and NO production in neutrophils. (a) NF-*κ*B DNA-binding activity. (b) iNOS mRNA expression as assessed by qPCR. (c) iNOS enzyme activity. (d) Assay of nitrite levels as an indicator of NO generation. The neutrophils were treated as shown in Figure 1(b). Data represent means ± SD for triplicates of each group.  ^#^ *P* < 0.05  versus ZY + vehicle (ZY).  ^§^ *P* < 0.05  versus ZY + ISO. NAI, NF-*κ*B activation inhibitor. DMSO is provided as a solvent control for NAI.

Based on the above findings, neutrophils are likely to represent a major component of ZY-induced inflammatory response in the mouse lung. To determine whether inflammatory mediators are modulated in neutrophils, we tested whether 0.7% ISO inhibited ZY-induced NF-*κ*B activation, iNOS activity, and NO generation. Consistent with the pulmonary tissue experiments (Figures 4 and 6), NF-*κ*B DNA-binding activity, iNOS activity, and iNOS-derived NO increased after ZY administration compared with control groups but were significantly attenuated by ISO treatment (*P* < 0.05; Figures 8(a)–8(d)). ISO + NAI treatment further inhibited iNOS activity and NO generation in ZY-stimulated neutrophils compared to a ISO treatment alone (Figures 8(b)–8(d)). The data suggests that ISO treatment inhibits ZY-induced NF-*κ*B and iNOS activation in neutrophils *in vitro* and that NF-*κ*B mediates the induction of iNOS gene expression and activity. 

## 4. Discussion

Volatile anesthetic ISO has been shown to exhibit anti-inflammatory effects. Prospects for clinical usage of ISO have been hampered due to adverse systemic effects. However, our recent report showed that subanesthetic dose of ISO (0.7% ISO) protected against ZY-induced shock by upregulating antioxidant enzymes [18]. We extend our findings in this study and demonstrate that 0.7% ISO reduces the development of lung injury in mice challenged with ZY via anti-inflammatory activity.

We first examined the effect of ISO on the pathobiology of ZY-mediated vascular lung injury in mice. Our results show that administration of ISO following onset of ZY-induced inflammation significantly prolongs survival and reduces pulmonary vascular damage. ZY also increases two indices of lung injury, lung water content, and protein leakage. ISO postconditioning significantly attenuated these conditions. Moreover, ISO prevented a significant loss of blood PaO_2_, PCO_2_,  HCO_3_^−^, and pH levels in the ZY-challenged mice, further suggesting improved lung function. Our studies also examined levels of enhancement by ZY of key proinflammatory cytokines (TNF-*α*, IL-1*β*, IL-6, and HMGB-1) in the BALF. ISO treatment was highly effective in reducing the enhancement of these proinflammatory cytokines.

During inflammation-related lung edema, endothelial and epithelial injuries are accompanied by an influx of neutrophils into the interstitium and bronchoalveolar space. The activation and transmigration of neutrophils play a key role in the progression of lung injury as seen in both clinical data and animal models [36, 37]. Neutrophil infiltration of the lung is controlled by a complex network of chemokines. In our study, subjects exposed to ZY alone exhibited markedly high levels of neutrophil chemoattractants (keratinocyte-derived chemokine, macrophage inflammatory protein-1*α*, macrophage inflammatory protein-2, and monocyte chemoattractant protein-1) in BALF, which was simultaneously associated with high MPO activity (an indicator of neutrophil infiltration) in pulmonary tissue. ISO attenuated the accumulation of these chemokines and downregulated MPO activity in the lung. Animal inflammation models also show that cell adhesion molecules (ICAM-1, VCAM-1, and E-selection) are associated with neutrophil infiltration in the lung [38]. Our studies suggest that ZY administration in mice upregulates mRNA expression of ICAM-1, VCAM-1, and E-selection in the lung. We also observed that ISO significantly decreased the expression of endothelial adhesion molecules E-selection, ICAM-1, and VCAM-1, consistent with previous studies [39, 40].

Inflammation results in increased activation of NF-*κ*B in the lungs [41]. In mouse models, induced neutropenia significantly diminishes the amount of NF-*κ*B that accumulates in the nuclei of pulmonary cell populations [38]. This indicates that neutrophils are important in modulating NF-*κ*B activation in the lungs. Experiments in mice show that, following endotoxin administration, nuclear concentrations of NF-*κ*B are increased in the neutrophils that accumulate in the lungs, compared with peripheral blood neutrophils [42]. This pattern of increased activation of lung versus peripheral blood neutrophils is consistent with that of cytokine expression, where expression of IL-1*β* and TNF-*α* is markedly increased in neutrophils isolated from the lungs after inflammatory stimulus. In addition, inhibition of NF-*κ*B activation prevents inflammation-induced increases in edema, neutrophil infiltration, and proinflammatory cytokine expression in the lungs [43]. Our studies showed that ZY induced NF-*κ*B activation in lung tissue and purified primary neutrophils by promoting I*κ*B-*α* degradation and nuclear translocation of NF-*κ*B p65. However, ISO treatment substantially blocked NF-*κ*B activation. Furthermore, ZY did not markedly increase lymphocytes, macrophages, and eosinophils but did increase total cells and neutrophils in the BALF. ISO treatment decreased total cells in the BALF mainly by inhibiting neutrophil recruitment. Our results suggest that ISO treatment suppresses ZY-activated NF-*κ*B in the lung, where neutrophils are likely the major source of NF-*κ*B.

Enhanced formation of NO after the induction of iNOS has been implicated in the pathogenesis of the inflammatory process associated with ZY-induced shock [44]. In this study, we demonstrated that ISO attenuated NO release, evaluated as nitrite levels in murine BALF from ZY-challenged mice. The effect on NO formation correlated with the inhibition of iNOS expression by ISO as demonstrated by western blot and immunohistochemistry. It has been demonstrated that NF-*κ*B mediates the induction of iNOS in several NO-producing cell types on the basis of studies with iNOS gene promoter constructs [45] or with inhibitors of NF-*κ*B [46]. Furthermore, the 5′-upstream sequence of the murine iNOS gene contains NF-*κ*B binding sites. Lipopolysaccharide treatment increases binding activity for the NF-*κ*B sites, which might indicate translocation of NF-*κ*B into the nucleus [47]. In our report, ZY also induced NF-*κ*B binding activity, iNOS expression and activity, as well as NO generation in neutrophils, which were all significantly attenuated by ISO treatment. NF-*κ*B activation inhibitor blocked this induction of iNOS and NO synthesis, suggesting that NF-*κ*B activation mediates the induction of iNOS in ZY-stimulated primary mouse neutrophils. Hence, these results cumulatively indicate that ISO reduces the development of lung injury caused by ZY by downregulating nuclear concentrations of NF-*κ*B in the lung neutrophils.

The final but most essential aspect in our study is its clinical implication. In contrast to the clinical doses of ISO preconditioning that have been traditionally utilized, we have administered a subanesthetic dose of ISO after the onset of inflammatory response caused by ZY, an IP model that can reproduce many characteristics of sepsis, and this subanesthetic dose effectively ameliorated lung injury in mice. Importantly, inhaling ISO at a concentration of less than 1% has been used in intensive care unit patients for facilitating mechanical ventilation [48]. Thus, the clinical relevance in our studies of administrating ISO after the ZY insult further enhances the appeal of this treatment modality. Taken together, our results demonstrate that a subanesthetic dose of ISO is beneficial for ZY-induced lung injury due to its anti-inflammatory actions. The present study of subanesthetic doses of ISO offers a new avenue for future translational and clinical research and holds promise for the development of new therapeutic approaches.

## 5. Conclusions

The present study demonstrated that the degree of ZY-induced murine lung damage is significantly attenuated by subanesthetic doses of ISO (0.7% ISO) postconditioning. Clearly, 0.7% ISO reduces dependent on its anti-inflammatory effects: (1) BALF exudate volume, W/D weight ratio, protein leakage, and histologic scores; (2) the expression and activities of proinflammatory signaling molecules NF-*κ*B and iNOS; (3) the BALF levels of proinflammatory cytokines and chemokines, the expression of several extravasation-associated proteins, and MPO activity, as well as total cell number in the lung mainly by inhibiting neutrophils recruitment; (4) ultimately, mortality rate of ZY-challenged mice. In accordance with *in vivo* observations, we found that 0.7% ISO also inhibited NF-*κ*B activation and reduced iNOS activity and NO formation in ZY-stimulated primary mouse neutrophils, and NF-*κ*B activation promoted iNOS expression and increased iNOS activity and NO generation. These *in vivo* and *in vitro* results indicate a novel pharmacological action by subanesthetic doses of ISO for anti-inflammation in the future.

## Figures and Tables

**Table 1:** Total and differential cell counts in bronchoalveolar lavage fluid (×10^5^ cells/mL).

	Sham + vehicle	Sham + ISO	ZY + vehicle	ZY + ISO	*P*
Total cells	1.35 ± 1.00	1.30 ± 1.01	8.63 ± 3.02*	2.10 ± 0.68^#^	<0.05
Neutrophils	0.05 ± 0.04	0.05 ± 0.03	7.38 ± 1.46*	1.8 ± 0.52^#^	<0.05
Lymphocytes	0.06 ± 0.07	0.03 ± 0.03	0.16 ± 0.15	0.14 ± 0.17	ns
Macrophages	1.01 ± 0.46	1.00 ± 0.50	1.22 ± 0.55	0.96 ± 0.60	ns
Eosinophils	0.02 ± 0.03	0.15 ± 0.02	0.03 ± 0.02	0.03 ± 0.01	ns

Values are expressed as the mean ± SD. *P < 0.05 versus sham + vehicle; ^#^ *P* < 0.05 versus ZY + vehicle; ns: nonsignificant.

## Abbreviations

BALF: Bronchoalveolar lavage fluid
ELISA: Enzyme-linked immunosorbent assay
HMGB-1: High-mobility group box-1
ICAM-1: Intercellular adhesion molecules-1
IL-1*β*: Interleukin-1*β*
IL-6: Interleukin-6
IL-10: Interleukin-10
iNOS: Inducible nitric oxide synthase
IP: Intraperitoneal
ISO: Isoflurane
KC: Keratinocyte-derived chemokine
mAb: Monoclonal antibody
MCP-1: Monocyte chemoattractant protein-1
MIP-1*α*: Macrophage inflammatory protein-1*α*
MIP-2: Macrophage inflammatory protein-2
MODS: Multiple-organ dysfunction syndrome
MPO: Myeloperoxidase
NAI: NF-*κ*B activation inhibitor
NF-*κ*B: Nuclear factor-*κ*B
NO: Nitric oxide
NS: Normal saline
pAb: Polyclonal antibody
PBS: Phosphate-buffered saline
PMNs: Polymorphonuclear neutrophils
RA: Room air
TLR-2: Toll-like receptor-2
TNF-*α*: Tumor necrosis factor-*α*
VCAM-1: Vascular adhesion molecules-1
W/D: Wet/Dry
ZIGI: Zymosan-induced generalized inflammation
ZY: Zymosan.

## References

[1] Martin GS, Mannino DM, Eaton S, Moss M (2003). The epidemiology of sepsis in the United States from 1979 through 2000. *New England Journal of Medicine*.

[2] Alberti C, Brun-Buisson C, Burchardi H (2002). Epidemiology of sepsis and infection in ICU patients from an international multicentre cohort study. *Intensive Care Medicine*.

[3] Goris RJ, Boekholtz WK, van Bebber IP (1986). Multiple-organ failure and sepsis without bacteria: an experimental model. *Archives of Surgery*.

[4] Cuzzocrea S, Costantino G, Mazzon E (1999). Protective effect of N-acetylcysteine on multiple organ failure induced by zymosan in the rat. *Critical Care Medcine*.

[5] Di Paola R, Mazzon E, Genovese T (2009). Ethyl pyruvate reduces the development of zymosan-induced generalized inflammation in mice. *Critical Care Medicine*.

[6] Young RE, Thompson RD, Larbi KY (2004). Neutrophil Elastase (NE)-deficient mice demonstrate a nonredundant role for NE in neutrophil migration, generation of proinflammatory mediators, and phagocytosis in response to zymosan particles *in vivo*. *Journal of Immunology*.

[7] Chaves HV, Ribeiro RA, de Souza AM (2011). Experimental model of zymosan-induced arthritis in the rat temporomandibular joint: role of nitric oxide and neutrophils. *Journal of Biomedicine and Biotechnology*.

[8] Ikeda Y, Adachi Y, Ishii T, Miura N, Tamura H, Ohno N (2008). Dissociation of toll-like receptor 2-mediated innate immune response to zymosan by organic solvent-treatment without loss of dectin-1 reactivity. *Biological and Pharmaceutical Bulletin*.

[9] Fan J, Ye RD, Malik AB (2001). Transcriptional mechanisms of acute lung injury. *American Journal of Physiology-Lung Cellular and Molecular Physiology*.

[10] Crystal GJ, Malik G, Yoon S-H, Kim S-J (2012). Isoflurane late preconditioning against myocardial stunning is associated with enhanced antioxidant defenses. *Acta Anaesthesiologica Scandinavica*.

[11] Hofstetter C, Boost KA, Hoeg S (2007). Norepinephrine and vasopressin counteract anti-inflammatory effects of isoflurane in endotoxemic rats. *International Journal of Molecular Medicine*.

[12] Li QF, Zhu YS, Jiang H, Xu H, Sun Y (2009). Isoflurane preconditioning ameliorates endotoxin-induced acute lung injury and mortality in rats. *Anesthesia and Analgesia*.

[13] Reutershan J, Chang D, Hayes JK, Ley K (2006). Protective effects of isoflurane pretreatment in endotoxin-induced lung injury. *Anesthesiology*.

[14] de Rossi LW, Brueckmann M, Rex S, Barderschneider M, Buhre W, Rossaint R (2004). Xenon and isloflurane differentially modulate lipopolysaccharide-induced activation of the nuclear transcription factor *κ*B and production of tumor necrosis factor-*α* and interleukin-6 in monocytes. *Anesthesia and Analgesia*.

[15] Si SJ, Fi FT, Ti CA, Cohn LH, Edmunds LH (2003). Cardiac anesthesia. *Cardiac Surgery in the Adult*.

[16] Sackey PV, Martling C, Carlswärd C, Sundin Ö, Radell PJ (2008). Short- and long-term follow-up of intensive care unit patients after sedation with isoflurane and midazolam-A pilot study. *Critical Care Medicine*.

[17] Shankar V, Churchwell KB, Deshpande JK (2006). Isoflurane therapy for severe refractory status asthmaticus in children. *Intensive Care Medicine*.

[18] Mu J, Xie K, Hou L (2010). Subanesthetic dose of isoflurane protects against zymosan-induced generalized inflammation and its associated acute lung injury in mice. *Shock*.

[19] Li J, Wang H, Li W (2013). Anesthetic isoflurane posttreatment attenuates experimental lung injury by inhibiting inflammation and apoptosis. *Mediators of Inflammation*.

[20] Boxio R, Bossenmeyer-Pourié C, Steinckwich N, Dournon C, Nüße O (2004). Mouse bone marrow contains large numbers of functionally competent neutrophils. *Journal of Leukocyte Biology*.

[21] Lin TN, He YY, Wu G, Khan M, Hsu CY, Marmarou A (1993). Effect of brain edema on infarct volume in a focal cerebral ischemia model in rats. *Stroke*.

[22] Chen J, Li Y, Wang L (2001). Therapeutic benefit of intravenous administration of bone marrow stromal cells after cerebral ischemia in rats. *Stroke*.

[23] Komotar RJ, Kim GH, Sughrue ME (2007). Neurologic assessment of somatosensory dysfunction following an experimental rodent model of cerebral ischemia. *Nature Protocols*.

[24] Clark JB, Nicklas WJ (1970). The metabolism of rat brain mitochondria. Preparation and characterization. *Journal of Biological Chemistry*.

[25] Ashwal S, Tone B, Tian HR, Cole DJ, Pearce WJ (1998). Core and penumbral nitric oxide synthase activity during cerebral ischemia and reperfusion. *Stroke*.

[26] Li J, Liu W, Ding S (2008). Hyperbaric oxygen preconditioning induces tolerance against brain ischemia-reperfusion injury by upregulation of antioxidant enzymes in rats. *Brain Research*.

[27] Ye R, Kong X, Yang Q, Zhang Y, Han J, Zhao G (2011). Ginsenoside Rd attenuates redox imbalance and improves stroke outcome after focal cerebral ischemia in aged mice. *Neuropharmacology*.

[28] Barrientos A, Kenyon L, Moraes CT (1998). Human xenomitochondrial cybrids: cellular models of mitochondrial complex I deficiency. *Journal of Biological Chemistry*.

[29] Dave KR, DeFazio RA, Raval AP (2008). Ischemic preconditioning targets the respiration of synaptic mitochondria via protein kinase C*ε*. *Journal of Neuroscience*.

[30] Krahenbuhl S, Chang M, Brass EP, Hoppel CL (1991). Decreased activities of ubiquinol: ferricytochrome c oxidoreductase (complex III) and ferrocytochrome c:oxygen oxidoreductase (complex IV) in liver mitochondria from rats with hydroxycobalamin[c-lactam]-induced methylmalonic aciduria. *Journal of Biological Chemistry*.

[31] Soper JW, Pedersen PL (1979). Isolation of an oligomycin-sensitive ATPase complex from rat liver mitochondria. *Methods in Enzymology C*.

[32] Kelly MM, McNagny K, Williams DL (2008). The lung responds to zymosan in a unique manner independent of Toll-like receptors, complement, and dectin-1. *American Journal of Respiratory Cell and Molecular Biology*.

[33] Byung HK, Seong SH, Soon WK (2008). Diarctigenin, a lignan constituent from Arctium lappa, down-regulated zymosan-induced transcription of inflammatory genes through suppression of DNA binding ability of nuclear factor-*κ*B in macrophages. *Journal of Pharmacology and Experimental Therapeutics*.

[34] Grommes J, Alard J, Drechsler M (2012). Disruption of platelet-derived chemokine heteromers prevents neutrophil extravasation in acute lung injury. *American Journal of Respiratory and Critical Care Medicine*.

[35] Choudhury S, Wilson MR, Goddard ME, O’Dea KP, Takata M (2004). Mechanisms of early pulmonary neutrophil sequestration in ventilator-induced lung injury in mice. *American Journal of Physiology-Lung Cellular and Molecular Physiology*.

[36] Abraham E (2003). Neutrophils and acute lung injury. *Critical Care Medicine*.

[37] Steinberg KP, Milberg JA, Martin TR, Maunder RJ, Cockrill BA, Hudson LD (1994). Evolution of bronchoalveolar cell populations in the adult respiratory distress syndrome. *American Journal of Respiratory and Critical Care Medicine*.

[38] Abraham E, Carmody A, Shenkar R, Arcaroli J (2000). Neutrophils as early immunologic effectors in hemorrhage- or endotoxemia-induced acute lung injury. *American Journal of Physiology-Lung Cellular and Molecular Physiology*.

[39] de Rossi LW, Horn NA, Buhre W, Gass F, Hutschenreuter G, Rossaint R (2002). The effect of isoflurane on neutrophil selectin and *β*2-integrin activation *in vitro*. *Anesthesia and Analgesia*.

[40] Biao Z, Zhanggang X, Hao J, Changhong M, Jing C (2005). The *in vitro* effect of desflurane preconditioning on endothelial adhesion molecules and mRNA expression. *Anesthesia and Analgesia*.

[41] Segal BH, Han W, Bushey JJ (2010). NADPH oxidase limits innate immune responses in the lungs in mice. *PloS ONE*.

[42] Shenkar R, Abraham E (1999). Mechanisms of lung neutrophil activation after hemorrhage or endotoxemia: roles of reactive oxygen intermediates, NF-*κ*B, and cyclic AMP response element binding protein. *Journal of Immunology*.

[43] Blackwell TS, Blackwell TR, Holden EP, Christman BW, Christman JW (1996). *In vivo* antioxidant treatment suppresses nuclear factor-*κ*B activation and neutrophilic lung inflammation. *Journal of Immunology*.

[44] Cuzzocrea S, Filippelli A, Zingarelli B, Falciani M, Caputi AP, Rossi F (1997). Role of nitric oxide in a nonseptic shock model induced by zymosan in the rat. *Shock*.

[45] Xie Q, Kashiwabara Y, Nathan C (1994). Role of transcription factor NF-*κ*B/Rel in induction of nitric oxide synthase. *Journal of Biological Chemistry*.

[46] Nunokawa Y, Oikawa S, Tanaka S (1996). Human inducible nitric oxide synthase gene is transcriptionally regulated by nuclear factor-*κ*B dependent mechanism. *Biochemical and Biophysical Research Communications*.

[47] Kinugawa K, Shimizu T, Yao A, Kohmoto O, Serizawa T, Takahashi T (1997). Transcriptional regulation of inducible nitric oxide synthase in cultured neonatal rat cardiac myocytes. *Circulation Research*.

[48] L’Her E, Dy L, Pili R (2008). Feasibility and potential cost/benefit of routine isoflurane sedation using an anesthetic-conserving device: a prospective observational study. *Respiratory Care*.

